# Editorial: Technologies for diabetes, volume III

**DOI:** 10.3389/fendo.2026.1846249

**Published:** 2026-04-14

**Authors:** Roque Cardona-Hernandez, Davide Tinti, Maurizio Delvecchio

**Affiliations:** 1 Division of Pediatric Endocrinology, Hospital Sant Joan de Déu, Barcelona, Spain; 2 Pediatric Diabetology Unit, Azienda Ospedaliero Universitaria Città della Salute e della Scienza di Torino, Turin, Italy; 3 Department of Biotechnological and Applied Clinical Sciences, University of L’Aquila, L’Aquila, Italy

**Keywords:** artificial pancreas, automated insulin delivery, continuous glucose monitoring, diabetes mellitus, digital health, hybrid closed-loop system, insulin

Over the past two decades, the management of diabetes has undergone a profound transformation driven by rapid technological innovation (1, 2). Advances in continuous glucose monitoring (CGM), automated insulin delivery (AID) systems, digital health platforms, and novel pharmacological and therapeutic approaches have fundamentally reshaped how clinicians and patients approach glycemic control (3, 4). These technologies have not only improved metabolic outcomes but have also shifted diabetes care toward a more personalized patient-centered model driven by data (5).

Historically, diabetes management relied heavily on self-monitoring blood glucose monitoring with fingerstick and insulin adjustments, often resulting in significant glycemic variability and suboptimal control. The introduction of CGM systems marked a pivotal step forward by providing real-time glucose data, trend analysis, and predictive alerts (1). These innovations have enabled both patients and healthcare professionals to make more informed therapeutic decisions and have laid the groundwork for AID technologies (6). In parallel, digital health tools, telemedicine models, and artificial intelligence–driven algorithms are increasingly integrated into routine care, expanding access and enabling continuous monitoring beyond the clinical setting (7).

This Research Topic collected fourteen contributions that collectively highlight the expanding role of technology and innovation in diabetes care. The articles span multiple domains, including glucose monitoring technologies, AID systems, digital health models, novel insulin strategies, nutritional considerations in the context of advanced technologies, and emerging control algorithms for artificial pancreas systems. Together, these studies illustrate the multidimensional progress occurring across the field and provide valuable insights into how technological advances can improve both clinical outcomes and quality of life for people living with diabetes.

## Advances in CGM technologies

CGM has become a cornerstone of modern diabetes care, providing detailed information on glucose trends and variability that cannot be captured by traditional self-monitoring of blood glucose (2). In this context, the evaluation of new CGM devices remains essential to ensure accuracy, reliability, and clinical utility. In this Research Topic, Zhao et al. evaluated the clinical performance of the SiJoy GS1 continuous glucose monitoring system during oral glucose tolerance testing in healthy adults. The study provides valuable data regarding the accuracy and responsiveness of this novel CGM device under dynamic glycemic conditions. Such evaluations are critical as new sensors enter the market, particularly given the growing reliance on CGM data to guide therapeutic decisions and to drive AID algorithms. Beyond device validation, CGM is increasingly used as a research and clinical tool to assess treatment responses and optimize therapy. In this context, Kravos Tramsek et al. examined the switch from premixed insulin analogues to the fixed combination of insulin degludec and liraglutide, using CGM metrics to evaluate glycemic outcome. By leveraging CGM-derived parameters such as time in range and glycemic variability, the authors show how modern monitoring technologies can provide a more nuanced assessment of therapeutic efficacy compared with traditional metrics alone.

## Automated insulin delivery and hybrid closed-loop systems

The most transformative technological development in diabetes care has been the emergence of AID systems, commonly referred to as hybrid closed-loop (HCL) or advanced hybrid closed-loop (AHCL) systems (4). These platforms integrate CGM sensors with insulin pumps and algorithm-driven control systems to automate insulin dosing and maintain glucose levels within target ranges.

Several studies in this Research Topic explore the real-world performance and clinical impact of such systems. The one-year real-world evaluation of advanced hybrid closed-loop systems in young children with type 1 diabetes by Franzone et al. provides important evidence regarding the safety and effectiveness of these technologies in young patients. Managing type 1 diabetes in these individuals presents unique challenges, including unpredictable eating patterns and high insulin sensitivity. The findings highlight how automated systems can significantly improve glycemic outcomes while reducing the burden on caregivers.

Complementing these findings, interestingly Mameli et al. illustrate the potential benefits of early technological intervention through the use of an AHCL system in a child with early-identified type 1 diabetes. The report emphasizes not only the safety and feasibility of these systems but also their potential role in optimizing glycemic management soon after diagnosis.

Further evidence supporting the effectiveness of AID comes from specific studies evaluating commercial systems. Bassi et al. assess the real-world efficacy of recommended MiniMed™ 780G settings in youth and young adults with type 1 diabetes, demonstrating improvements in time in range and overall glycemic metrics when standardized algorithm settings are applied. This article follows the wave of successful previous publications from the same country addressing the same device (8, 9). Another study group examines in an observational study the glycemic outcomes associated with a tubeless automated insulin delivery system in a real-world cohort (Angelino et al.). Tubeless systems represent an important advancement by improving comfort and reducing device burden, potentially increasing adherence and acceptance among patients.

Together, these studies underscore the rapid maturation of AID technologies and highlight their growing role in routine clinical practice.

## Technological innovations in insulin therapy

In addition to advances in monitoring and automation, technological innovation is also influencing how insulin itself is delivered. One study investigates the efficacy and safety of needle-free injection systems in patients with type 2 diabetes undergoing intensive insulin therapy (Wang et al.). Needle-free technologies may help address barriers such as injection discomfort, needle anxiety, and adherence challenges, potentially improving long-term treatment outcomes.

Wang et. explore the effects of short-term intensive insulin pump therapy on serum endotrophin levels in newly diagnosed type 2 diabetes (Li et al.). Although the primary focus is metabolic and biochemical, the study reflects the broader trend of integrating technological approaches (insulin pump) into earlier stages of diabetes management.

Additionally, the article discussing practical considerations for using concentrated U200 insulin in automated insulin delivery systems (Marks et al.) addresses an emerging clinical need. As insulin requirements vary widely among individuals, adapting automated systems to accommodate different insulin formulations may expand their applicability to patients with higher insulin demands.

## Digital health and hybrid care models

The digital transformation of healthcare is also profoundly affecting diabetes management. Telemedicine platforms, remote monitoring tools, and digital engagement strategies are increasingly incorporated into clinical care, enabling continuous interaction between patients and healthcare teams (7).

An illustrative example is the study by Zakaria et al. evaluating the impact of virtual patient engagement on glycemic control within the GluCare hybrid care model. This retrospective observational analysis demonstrates how integrating digital communication, remote monitoring, and multidisciplinary care can lead to improved metabolic outcomes in individuals with type 2 diabetes. Such models have become particularly relevant in the context of expanding telehealth adoption and the need for scalable solutions to manage the growing global burden of diabetes.

## Nutritional and pharmacological considerations in the era of diabetes technology

Nutrition and pharmacotherapy are affected by technological tools as well. Talbo et al. explore nutrition therapy in the era of AID. Automated systems can mitigate some of the challenges associated with carbohydrate counting and meal-related glucose excursions, but nutrition remains a fundamental component of diabetes care. The authors discuss how dietary strategies must evolve alongside technological innovations to optimize metabolic outcomes. On the other hand, Alhowiti and Mirghani examine the effects of GLP-1 receptor agonists on HbA1c and insulin dose in individuals with type 1 diabetes, highlighting the potential of combination therapies to enhance glycemic control and reduce insulin requirements. As new pharmacological agents emerge, drug and technological systems may interestingly interplay improving glucose control.

## Engineering and control algorithms for the artificial pancreas

Beyond clinical implementation, technological innovation in diabetes management also relies on advances in engineering and control theory. The development of sophisticated algorithms capable of predicting glucose dynamics and adjusting insulin delivery in real time is central to the evolution of artificial pancreas systems (10, 11).

Xinh et al. propose a backstepping control strategy for artificial pancreas systems based on sub-fixed-time stability theory (Xing et al.). This work represents an interesting example of interdisciplinary collaboration between engineering and medicine. By developing more robust and responsive control algorithms, such approaches may further enhance the safety and effectiveness of fully AID systems in the future.

## Metabolic outcomes and long-term perspectives

Finally, the Research Topic also includes a three-year follow-up study by Wu et al. exploring long-term metabolic outcomes beyond technology-driven interventions. They examine maintained diabetes remission among individuals with normal BMI following intermittent interventions (Wu et al.) and provide insights into the complex interplay between metabolic physiology, lifestyle interventions, and long-term disease trajectories. Although not strictly focused on technology, such research complements technological advances by highlighting broader strategies for achieving durable metabolic improvements.

## Future directions

Taken together, the articles included in this Research Topic highlight the remarkable progress occurring at the intersection of technology, clinical practice, and biomedical research in diabetes care. From improved CGM systems and AID platforms to digital care models and advanced control algorithms, innovation is rapidly reshaping how diabetes is managed across diverse populations.

Importantly, these developments are not occurring in isolation. The integration of multiple technological components, such as CGM sensors, insulin pumps, algorithm-driven control systems, digital health platforms, and pharmacological adjuncts, suggests that the future of diabetes care will increasingly rely on interconnected ecosystems rather than individual devices (4) (Xing et al.). Such systems have the potential to deliver personalized therapy by continuously adapting treatment to the unique physiological and behavioral patterns of each individual.

At the same time, several challenges remain. Ensuring equitable access to advanced technologies, addressing usability and training needs, maintaining data security and privacy, and integrating large volumes of real-world data into clinical decision-making frameworks will be critical priorities in the coming years.

The studies presented in this Research Topic provide compelling evidence that technological innovation continues to drive meaningful improvements in diabetes management. By combining advances in engineering, digital health, pharmacology, and clinical research, the field is moving steadily toward the long-standing goal of achieving optimal glycemic control with minimal burden for people living with diabetes.

We hope that the work gathered in this Research Topic will stimulate further research, foster interdisciplinary collaboration, and ultimately contribute to the development of more effective, accessible, and patient-centered strategies for diabetes care.
